# Supplementary material for: Evolution and transmission dynamics of wild poliovirus in Pakistan and Afghanistan (2012-2023)
Source: Nat Commun. 2025 Jun 4;16:5170. doi: 10.1038/s41467-025-60432-x (PMC12137544; doi:10.1038/s41467-025-60432-x)
Supplement: Supplementary file 1 — Supplementary Information [file 41467_2025_60432_MOESM1_ESM.pdf]

Supplementary Text

Outlying sequences

The root-to-tip regression performed prior to phylogeographic analysis of the included sequence data showed a single outlying sequence. This was collected in 2020 in South Punjab region (PAK20-ENV-BMS422RD) and was excluded from the phylogenetic analyses as it was found to be most closely related to sequences in the Y cluster despite being previously assigned to the X cluster. This sequence was also found to be an outlier from the clock rate of 0.011 substitutions per site per year (red point in Fig. S8) estimated based on all included sequence data which is in line with previously reported WPV1 VP1 clock rates.[17]

Tip-state-swap phylogeographic analysis

The model uses the 'TipStateSwapOperator' operator in BEAST to uniformly resample the locations at the tips of the phylogeny at each iteration to give a null prior distribution of movements between the locations.[22,23] This null model is informed by the geographic sample used in the analysis but not the genetic data. The null model places the root of the full phylogeny in Sindh (pp = 0.156) and the root of all recent detections) when excluding early samples which appear to fall into a separate ancestral clade) in the south corridor region of Pakistan (pp =0.164). Both ancestors are found to be most probably in the north corridor region of Pakistan (pp = 0.255 and 0.615 respectively) in our analysis. This difference suggests that the locations of these ancestors is informed by the genetic data rather than biased sampling by location. We see the same difference for the ancestor of the clade B (tip-state-swap ancestor: North Corridor PK, pp = 0.192, inferred ancestor North Corridor AF, pp = 0.989) labelled in figure 2. For clade A the null and inferred ancestral locations are both in Karachi, although the posterior probability is much higher in our analysis (tip-state-swap pp = 0.316, inferred pp = 0.883).

The null model provided by the tip-state-swap phylogeographic analysis can also be used to correct the Bayes factors for the numbers of movements between regions inferred in the analysis. By correcting the Bayes factors using the posterior probabilities of each transition pathway under the tip-state-swap and inference models, we can account for transitions where significance is heavily influenced by regionally biased sampling. With the corrected Bayes factors under the tip-state-swap phylogeographic approach 43 pairs of regions are found to have strongly significant (BF>=10) transitions informing the model. This is down from 66 found to be strongly significant without correcting the Bayes factors. Most significantly, nine transitions out of Karachi to other regions and six into Karachi are found to be explainable under the null model informed by the regional sampling rates as are five out of the North Corridor region of Pakistan and two into this region. These two regions have the most samples in the analysis, with 16.7% and 15.4% of the total samples coming from Karachi and the North Corridor of Pakistan respectively.

AFP data alone

Figure S3 shows the reconstructed movements between regions with AFP data alone, a priori considered less biased than ES sampling which is included in the full analysis. As mentioned in the main text, ES sites are targeted to specific locations and the distribution and catchment of the sites varies across regions. Stool samples, on the other hand, are taken from AFP cases and should therefore more closely follow real-world differences in the distribution of infected individuals in space.

To assess the sensitivity of our model results to the inclusion of ES data we have also analyzed AFP samples alone. As there are fewer stool samples this results in a lower overall number of movements between regions (n = 268, 95% CrI 252-282). The patterns seen are similar to the full analysis shown in figure 2, with clear A and B clusters visible on the tree with all recent detections linked to the B cluster and, as mentioned in the main text, all recent case detections have been limited to the North Corridor AF and Central Corridor PK regions (light blue and bright pink in figure S3).

Karachi remains the largest exporter of virus in this analysis (n = 65, 95% CrI 40-82), with the same major exporting regions for the most part (Fig. S3C) as in the main analysis (Fig 1G). The major importers also remain the same, although the largest importer without ES data is determined to be the North Corridor Pk region. The major change in these movement estimates is the East Pk region, which reports a large number of ES positive detections but rarely reports paralysis cases. This region goes from a major importer and exporter of virus in the main analysis, playing a major role in movement from northern to southern regions during outbreak periods (Fig. 2) to a minor importer and sink region with very few onwards transmissions in this analysis. This highlights the additional utility of ES data when attempting to reconstruct the movement of poliovirus, where paralysis is a very rare outcome (and therefore contacts of paralysis cases are also rare). Incorporating the biases in ES data in future analyses would be of interest to reconstructing corrected movement rates between regions.

Correcting for biased sampling of AFP cases

Although sampling of AFP cases is considered less biased than sampling of ES due to the severity of the outcome leading to healthcare-seeking behavior, there is evidence from non-polio AFP cases that there is still differential reporting across the region (Fig. S7). Non-polio AFP cases, expected to be recorded at a rate of at least two per 100000 people per year in endemic countries to meet WHO reporting guidelines, are paralysis cases not found to be due to polio in testing.[58,59] This background rate of non-polio paralysis cases should give an indication of the sensitivity of polio monitoring systems to detect paralysis in the population, without the confounding factor of differential polio transmission between regions. Although not a perfectly unbiased metric, due to other (non-polio) infectious causes of paralysis which may be differentially transmitted, this measure is routinely used as an indicator of AFP surveillance performance by the WHO.[60–62] To test the impact of differences in sampling between regions, we adjust the sampling probabilities at the tips of the phylogeny in a simple discrete trait framework. This process is explained in a preprint (Jorgensen et al. 2023) and is implemented in BEAST using existing methods.[20,40,63] Adjusting for non-polio AFP rates, averaged (mean) over the full time-period covered by the data and normalized to sup to 1 across regions, led to minor differences compared to the uncorrected model. The main differences were the increase in estimated exports from the city of Karachi to other regions and decreased exportations from the north corridor region. All corrected movement estimates fell within the 95% CrIs of the uncorrected estimates. This method could be expanded in the future to account for sampling changes over time in each region with an epoch model although further testing is required to ensure the correction is robust to these changes.

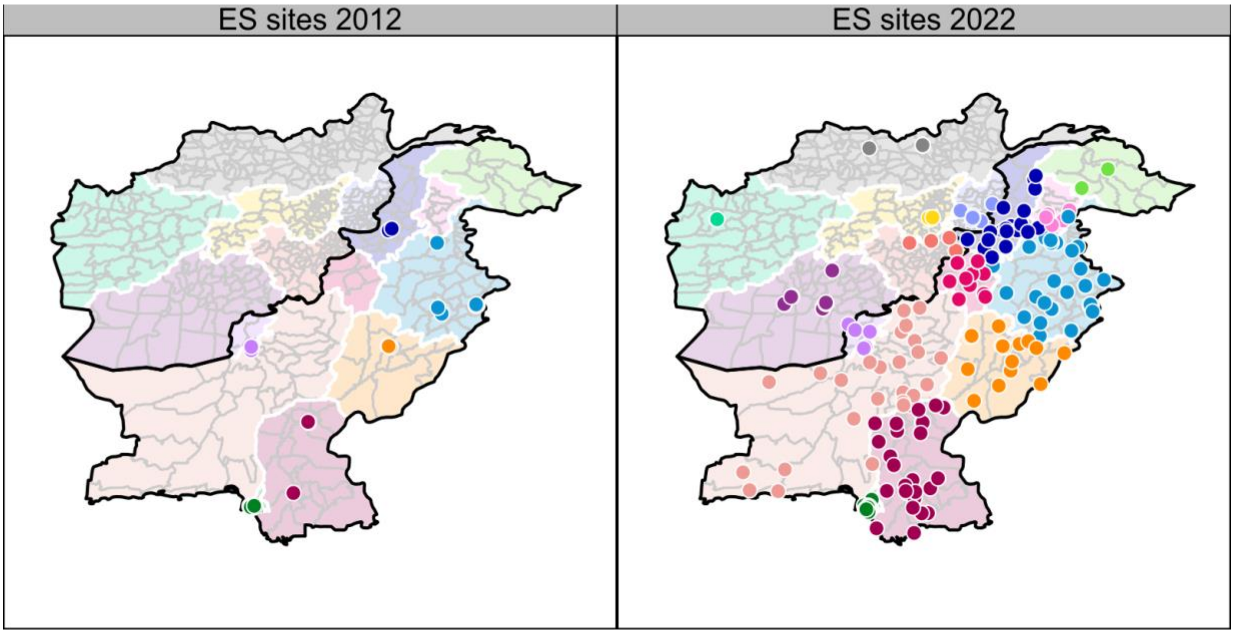

Fig. S1.

The number of environmental surveillance sites reporting samples in the calendar years 2012 and 2022 colored by region. The locations are approximate to the centroid of the district in which a site is located if definite coordinates are not available. This data is extracted from the POLIS database[9]. Geodata is sourced from the World Health Organization. The boundaries and names shown, and the designations used on the map do not imply the expression of any opinion whatsoever on the part of the authors or the World Health Organization concerning the legal status of any country, territory, city or area or of its authorities, or concerning the delimitation of its frontiers or boundaries.

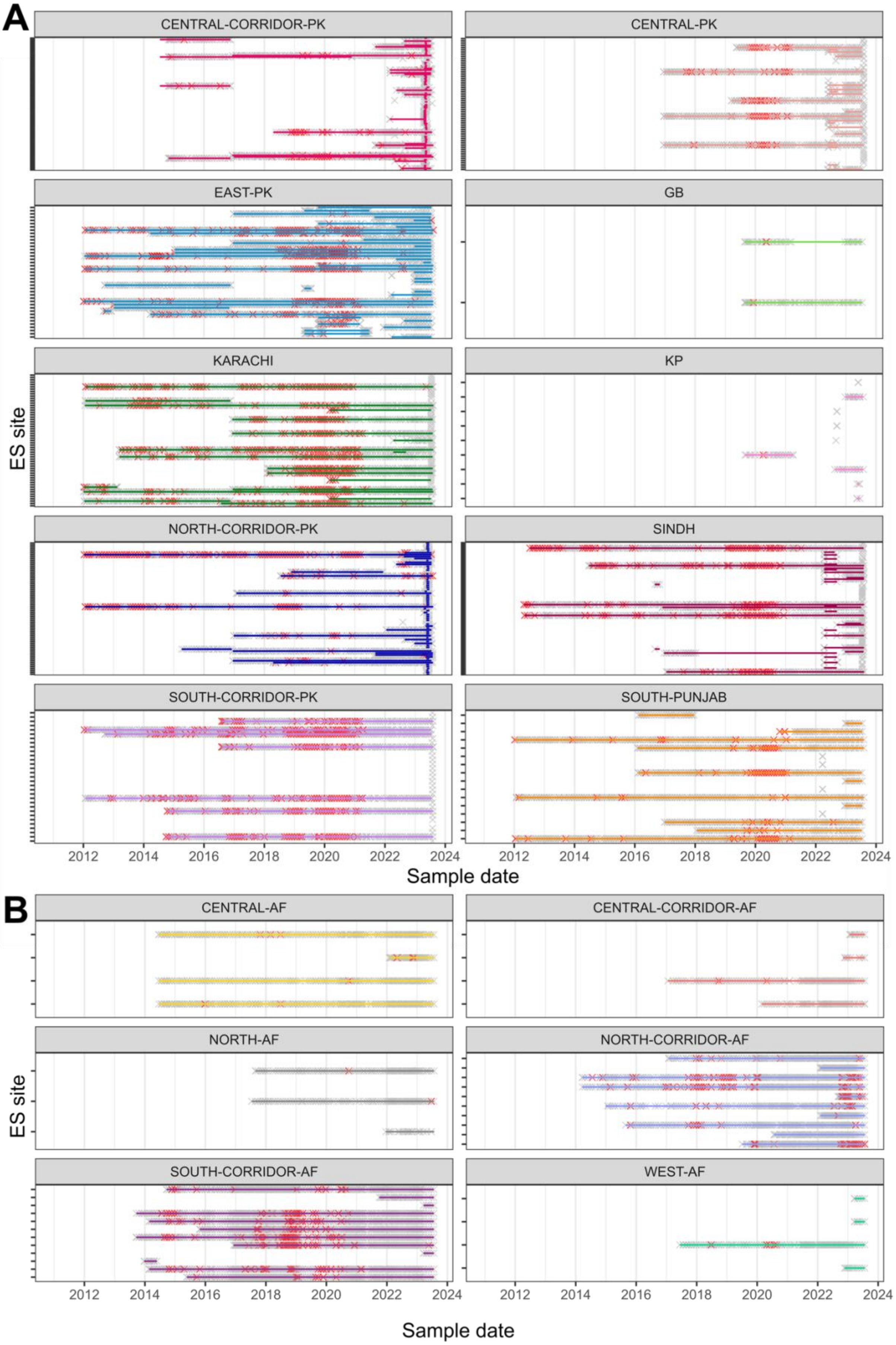
A
CENTRAL-CORRIDOR-PK
CENTRAL-PK
EAST-PK
GB
KARACHI
KP
NORTH-CORRIDOR-PK
SINDH
SOUTH-CORRIDOR-PK
SOUTH-PUNJAB
ES site
Sample date
B
CENTRAL-AF
CENTRAL-CORRIDOR-AF
NORTH-AF
NORTH-CORRIDOR-AF
SOUTH-CORRIDOR-AF
WEST-AF
ES site
Sample date
2012
2014
2016
2018
2020
2022
2024

Fig. S2.

Plots of the number of detections through time in each region of A) Pakistan and B) Afghanistan. Each cross represents a sample and each row a single sampling site. Red crosses are wild poliovirus detections, all other samples are shown in grey. Where there are multiple detections at the same site, they are linked with a horizontal line from first to last detection included in the dataset.

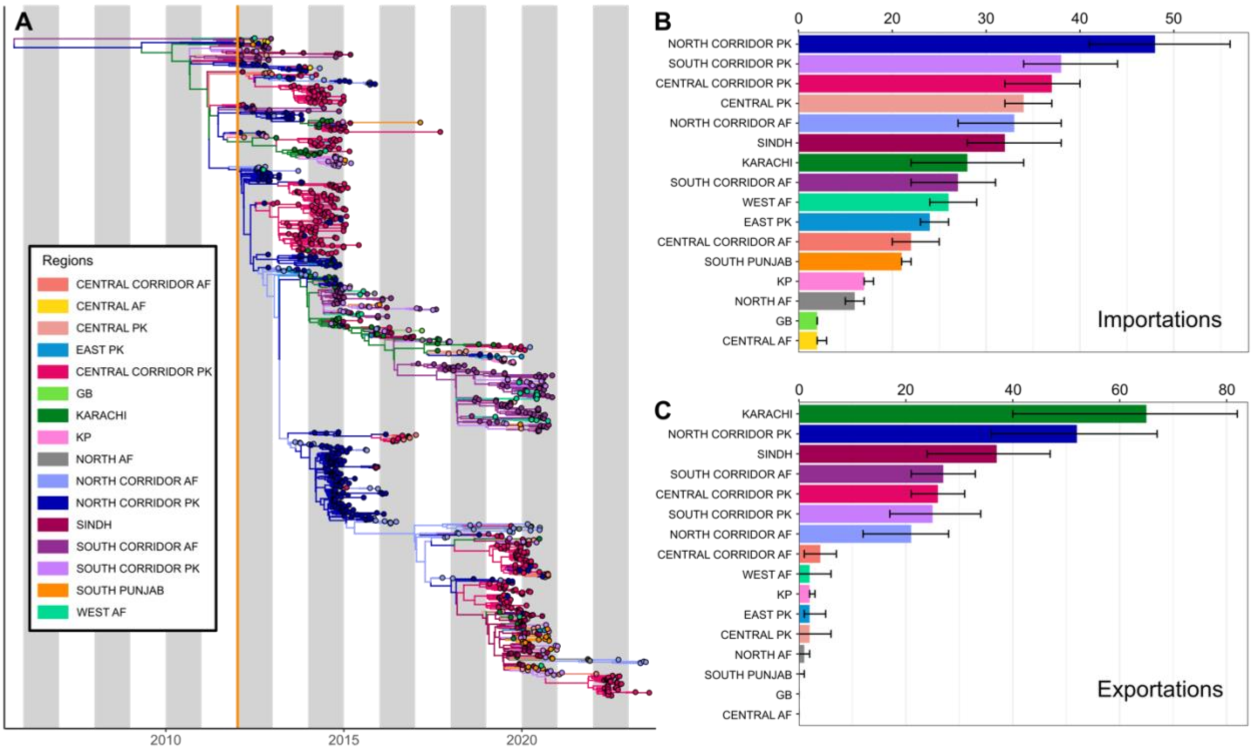

Fig. S3.

A) Phylogenetic tree of stool samples collected between January 2012 and August 2023. The vertical orange line represents the time of the first collected sequence. Branches and internal nodes are colored by their inferred location and tips by their known location. B) Inferred number of importations of virus by region over the full time period. C) Inferred numbers of exportations of virus by region over the full time period. Error bars on B and C represent the 95% credible interval of the estimated number of movements[43].

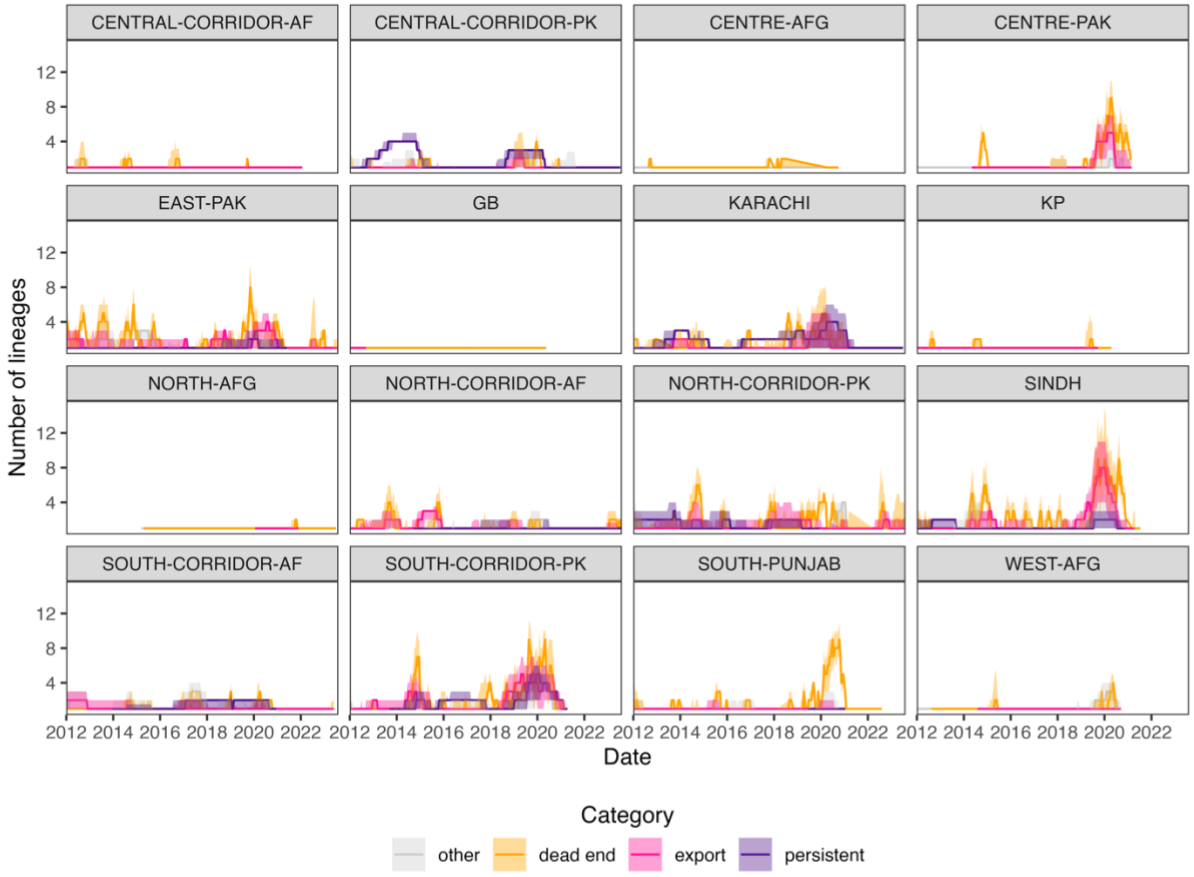

Fig. S4.
Median and 95% credible intervals for the number of LTLs falling into each classification across 300 phylogenies sampled from the posterior of the MJ Bayesian analysis. Median values and the method to designate these categories are shown in figure 4. Figure S6 shows an example set of LTLs on the consensus phylogeny from the same MJ analysis.

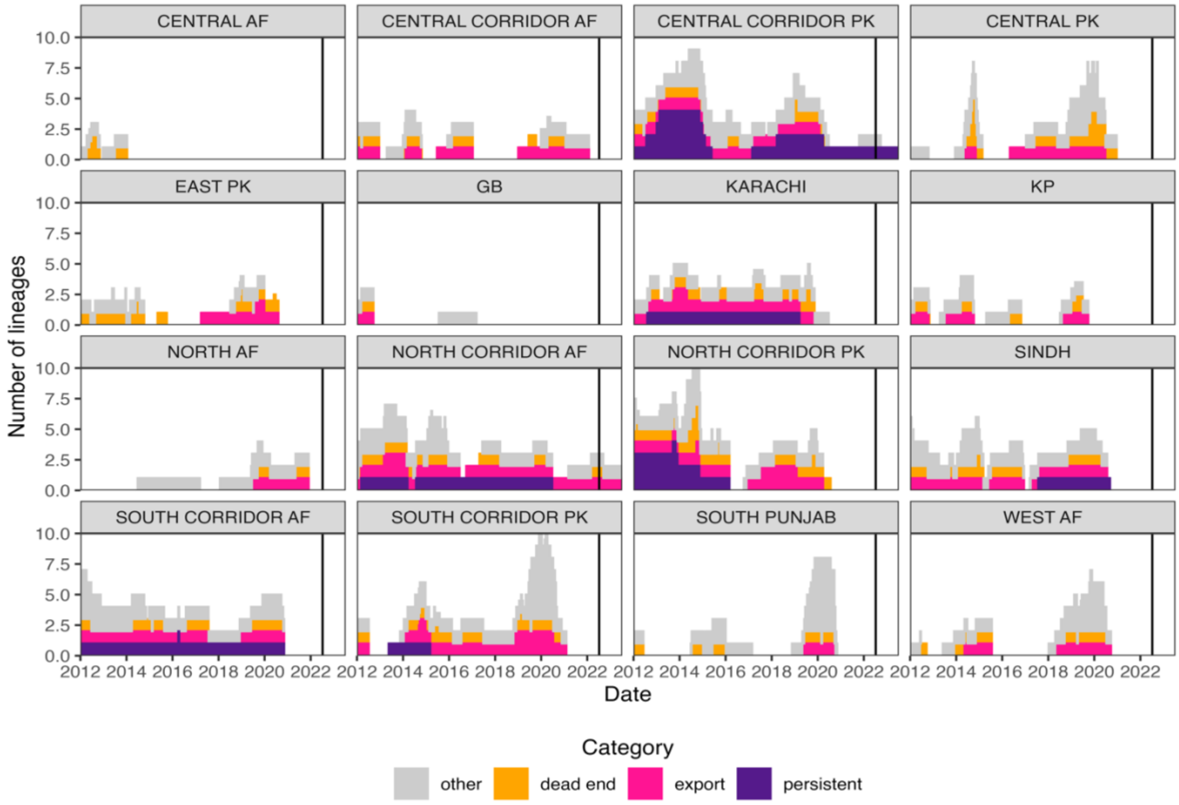

Fig. S5.
Median numbers of LTLs in each category over time using AFP data alone. Panels are split by the region in which the LTLs are reported. An example of how these lineages are generated and classified is provided in figure 4.

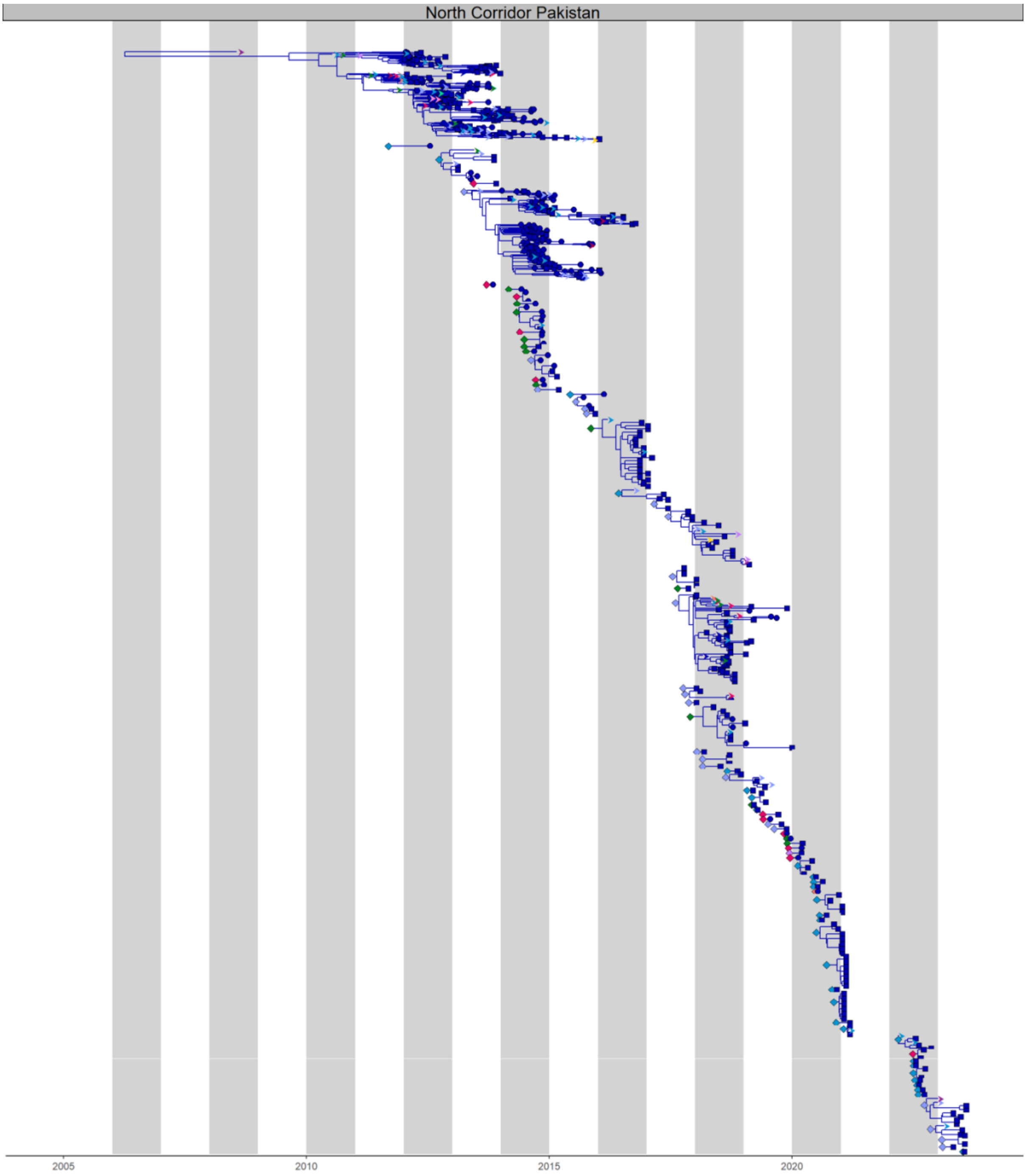

North Corridor Pakistan
2005
2010
2015
2020

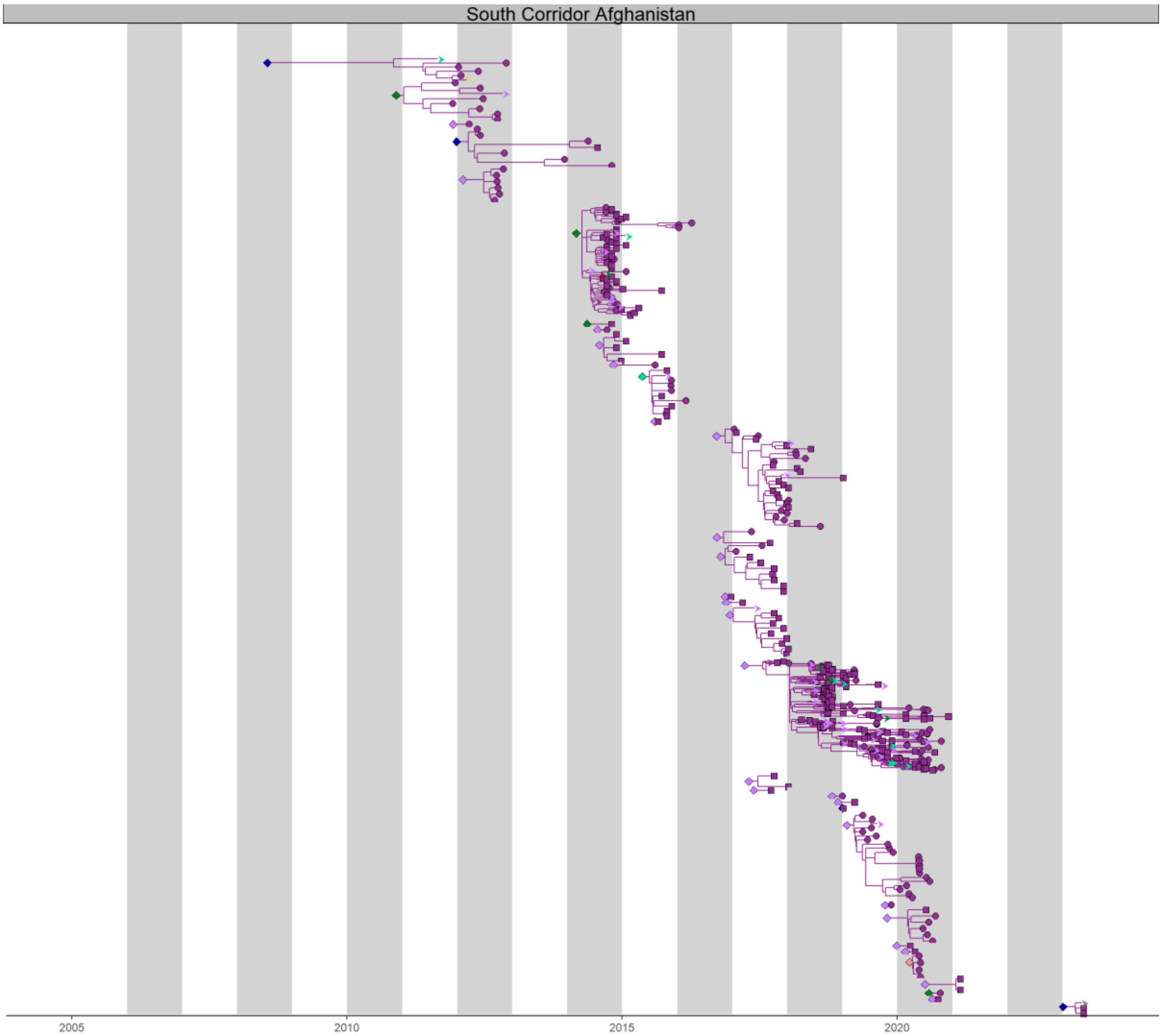
South Corridor Afghanistan
2005
2010
2015
2020

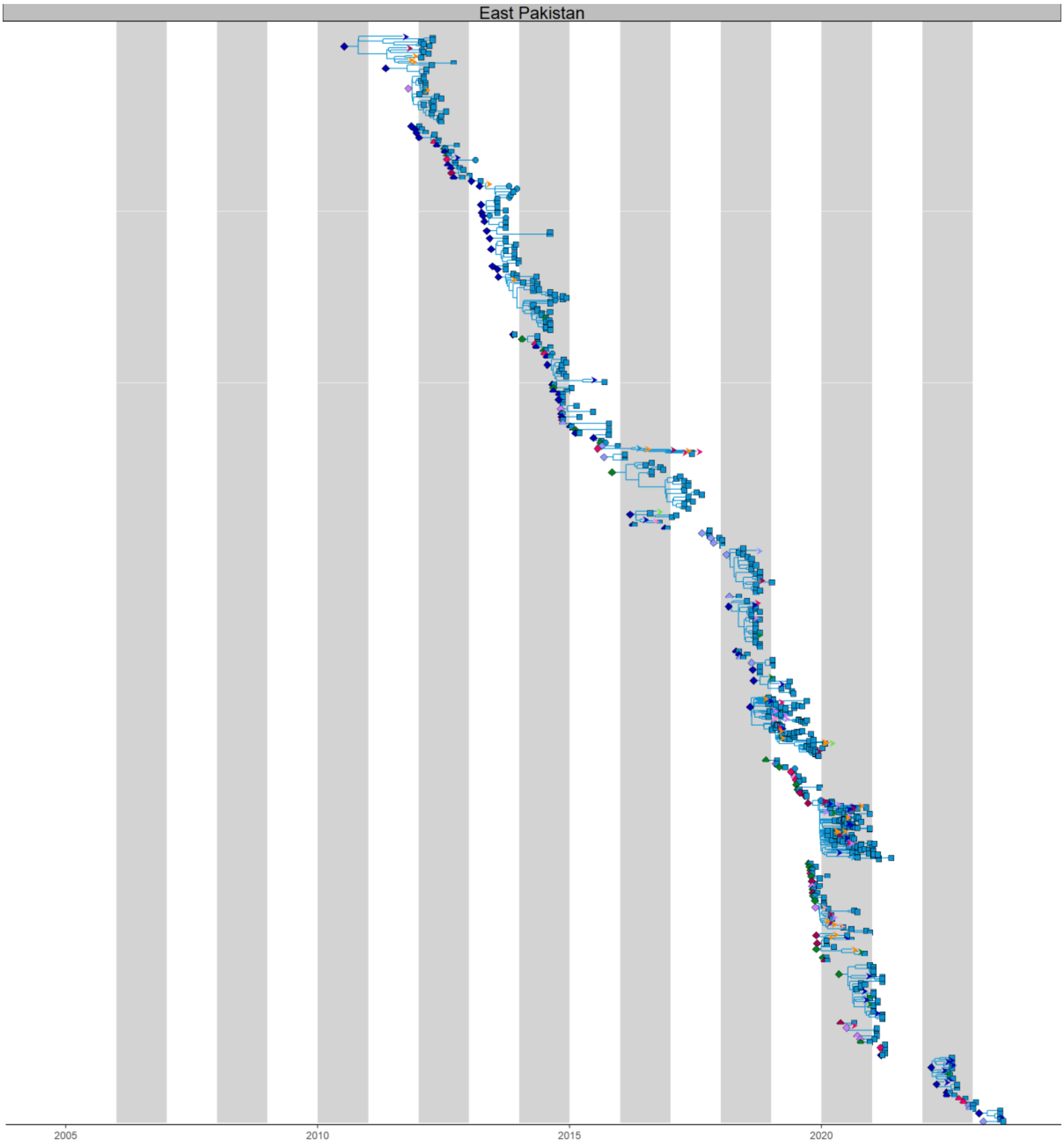
East Pakistan
2005
2010
2015
2020

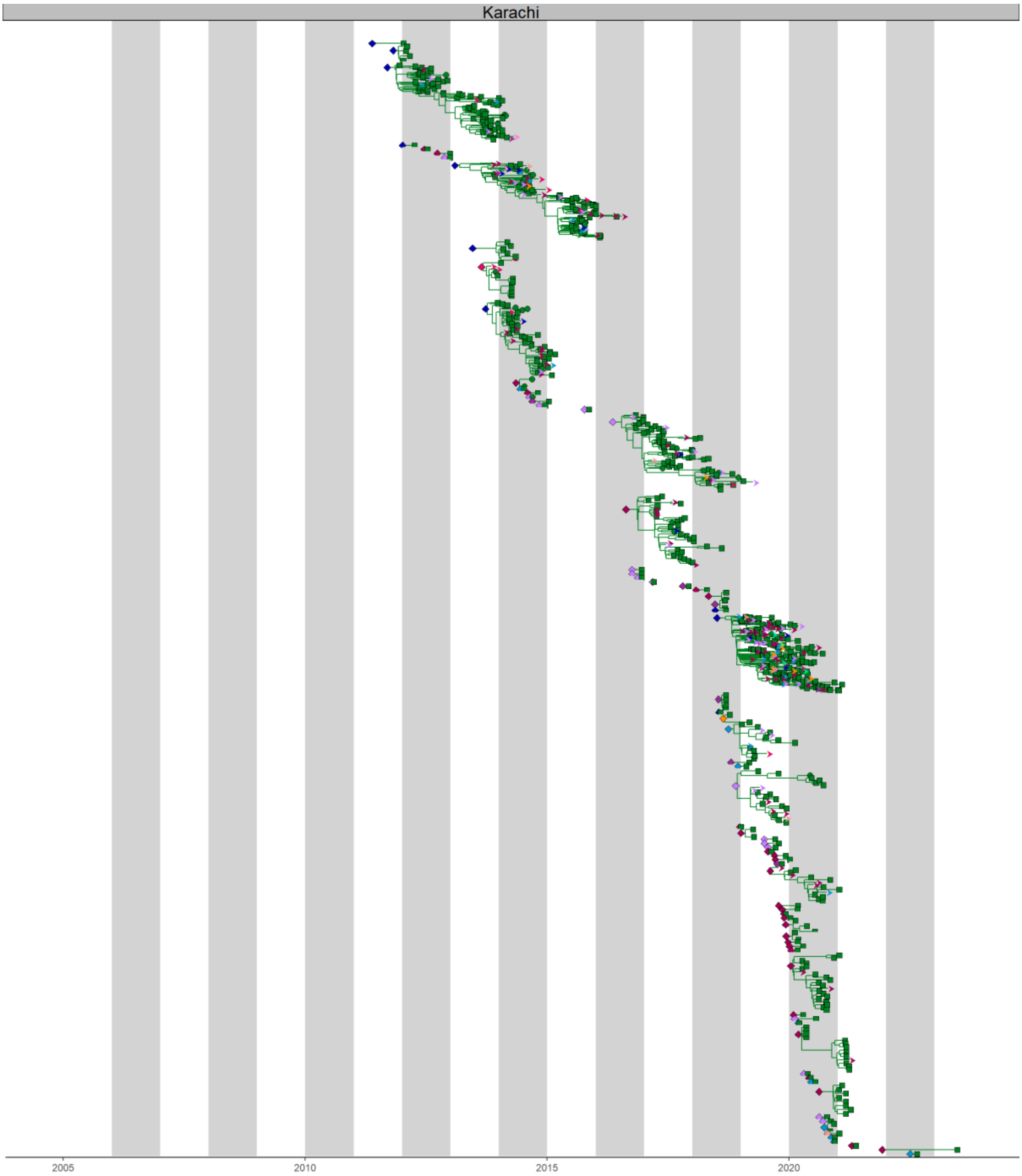
Karachi
2005
2010
2015
2020

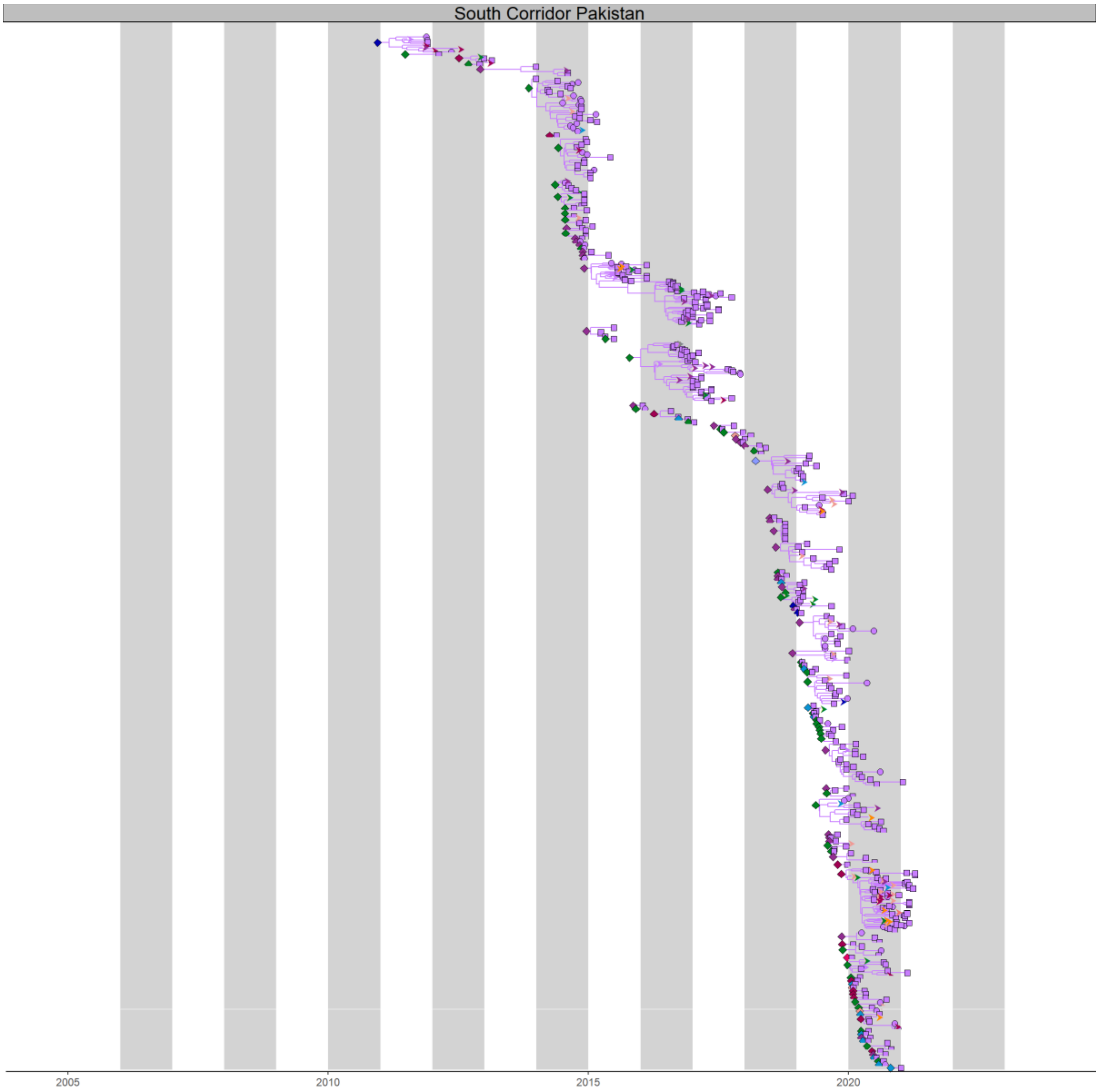
South Corridor Pakistan
2005
2010
2015
2020

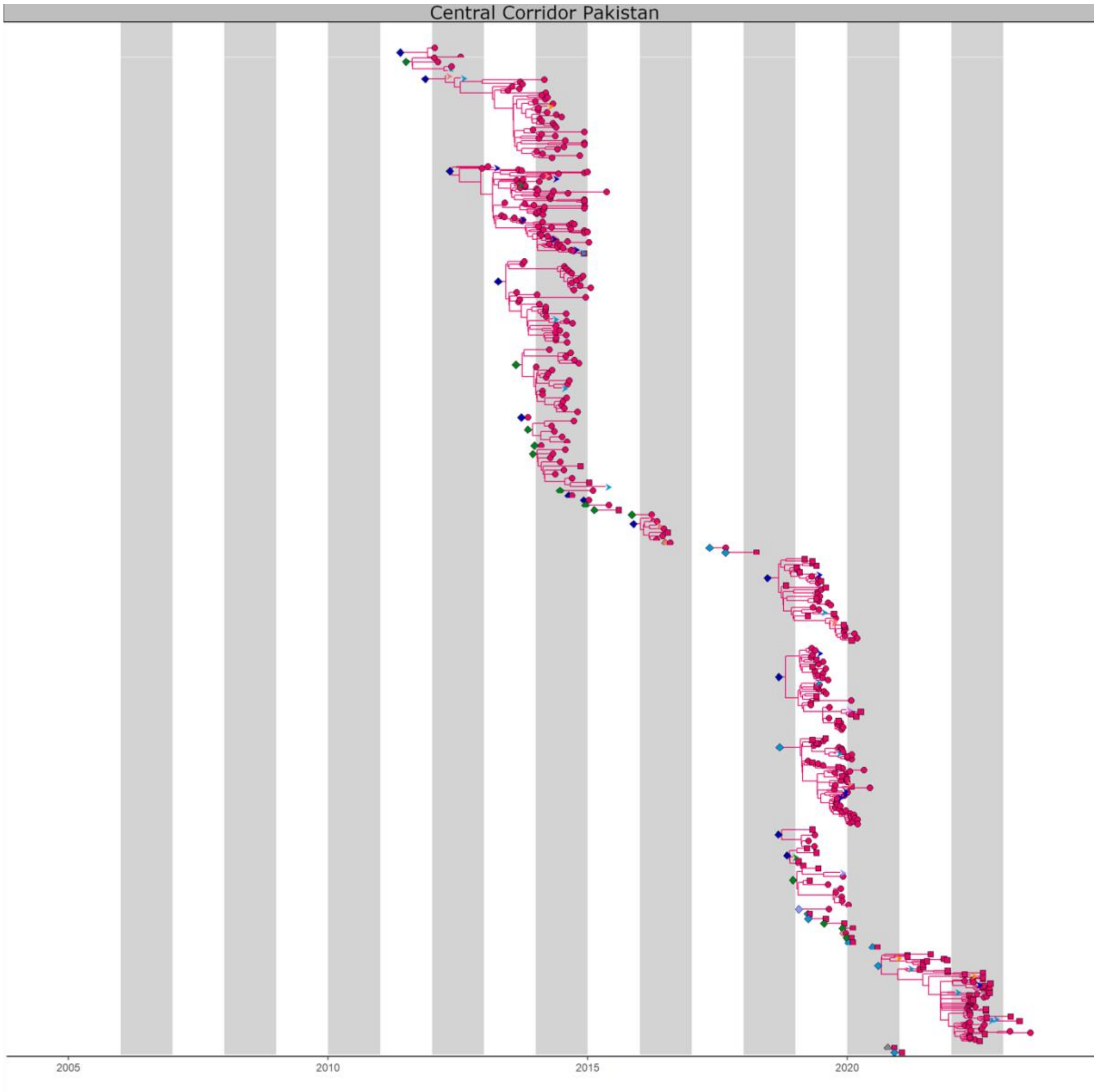

Central Corridor Pakistan
2005
2010
2015
2020

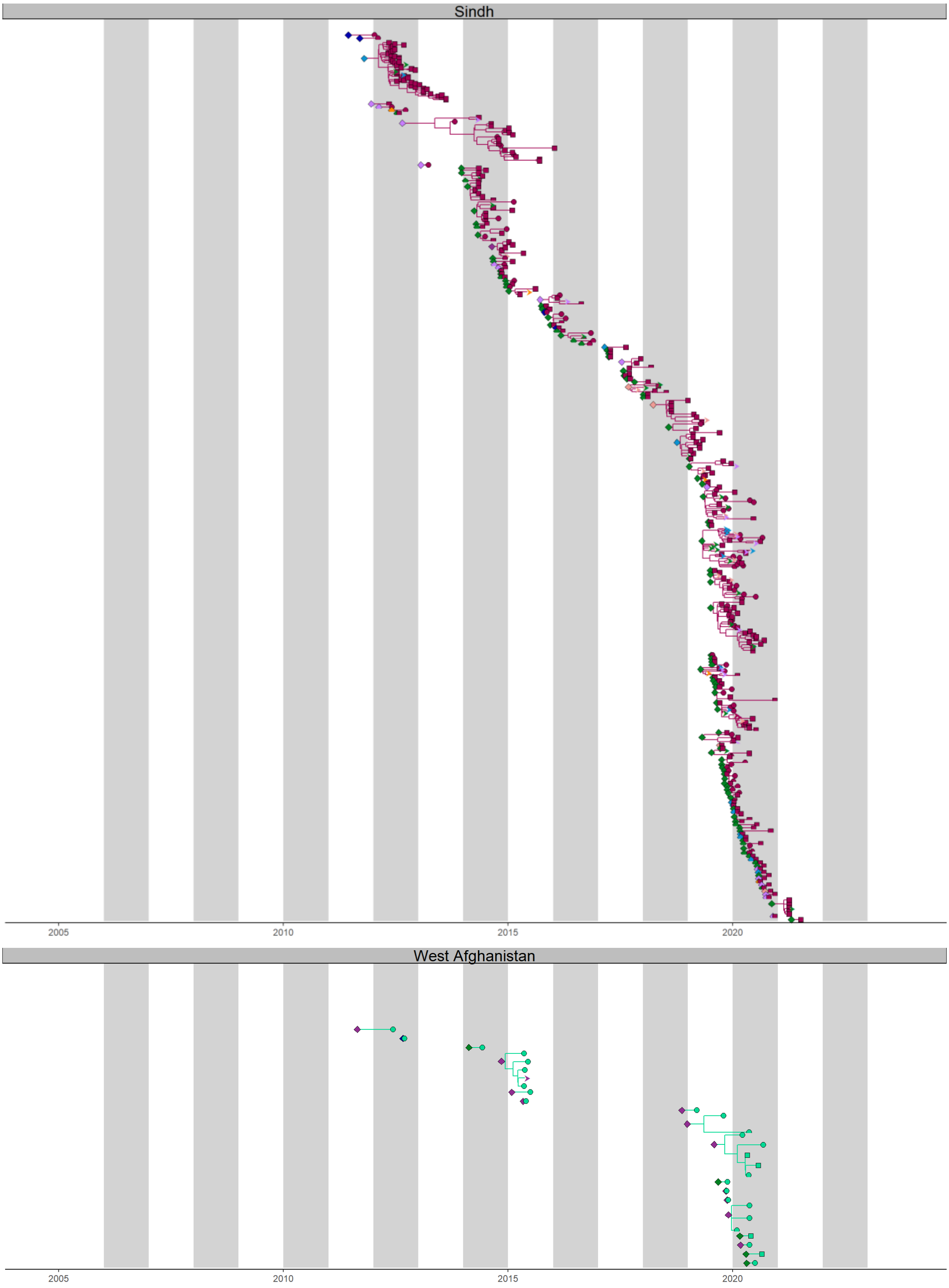
Sindh
2005
2010
2015
2020
West Afghanistan
2005
2010
2015
2020

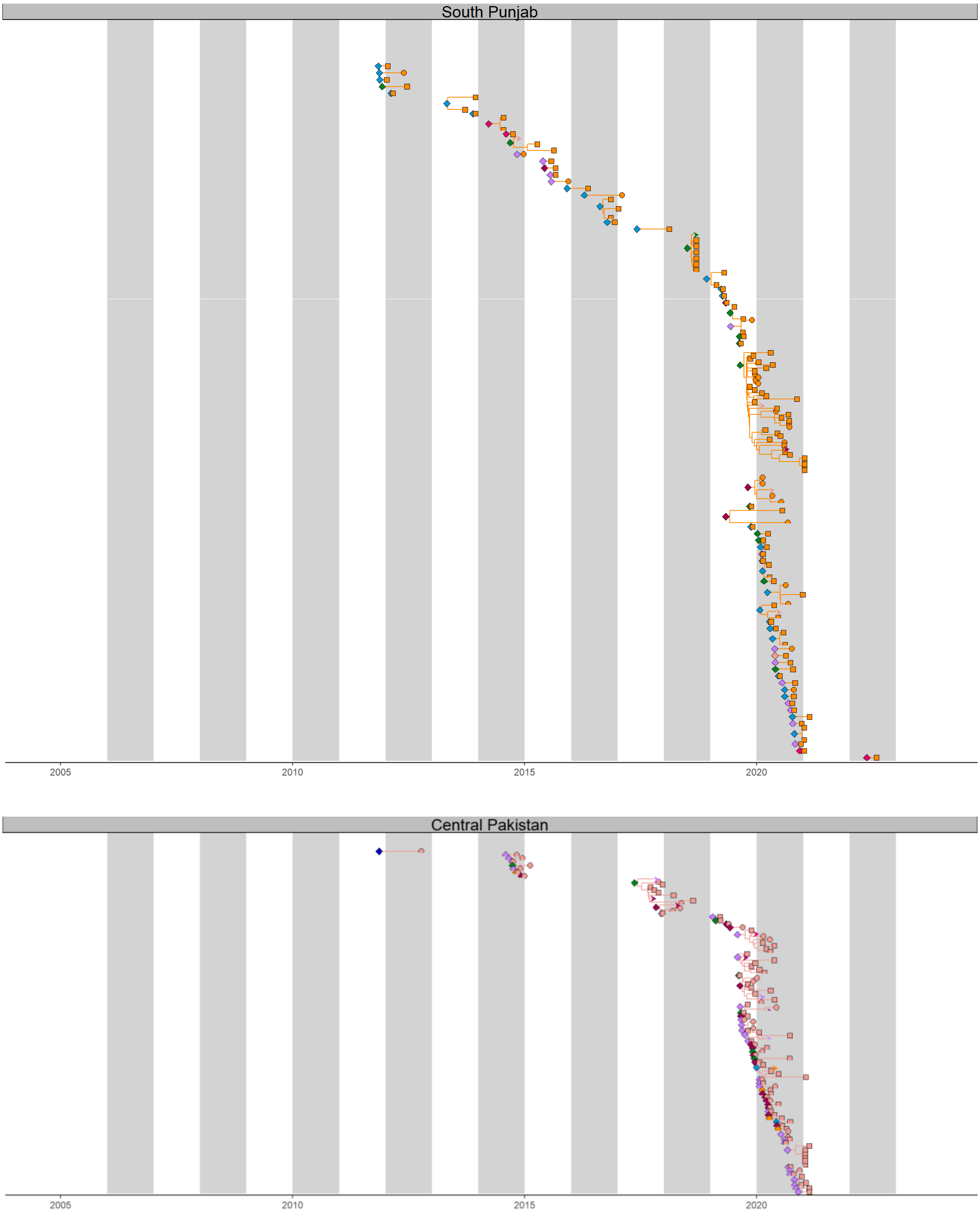
South Punjab
2005
2010
2015
2020
Central Pakistan
2005
2010
2015
2020

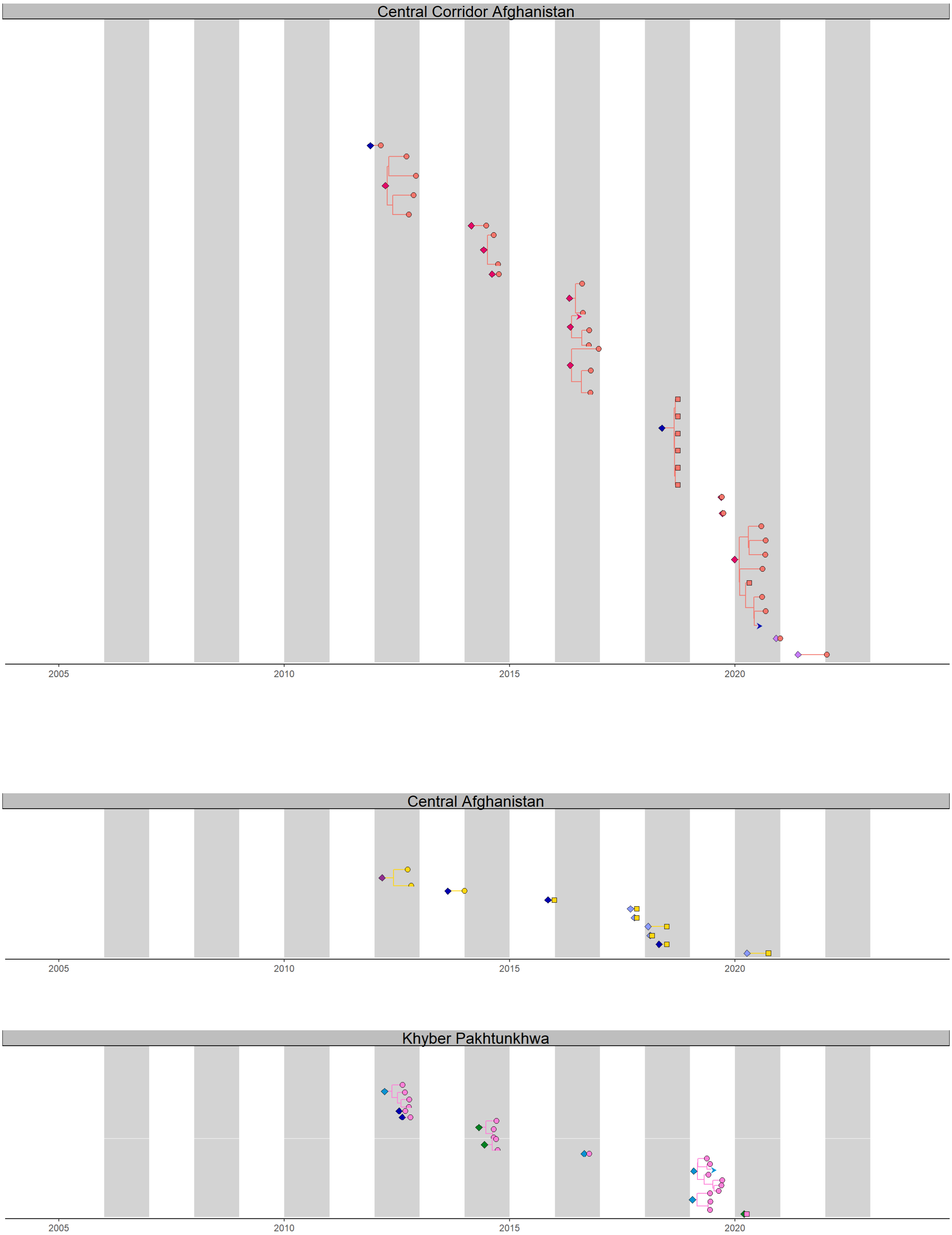
Central Corridor Afghanistan
2005
2010
2015
2020
Central Afghanistan
2005
2010
2015
2020
Khyber Pakhtunkhwa
2005
2010
2015
2020

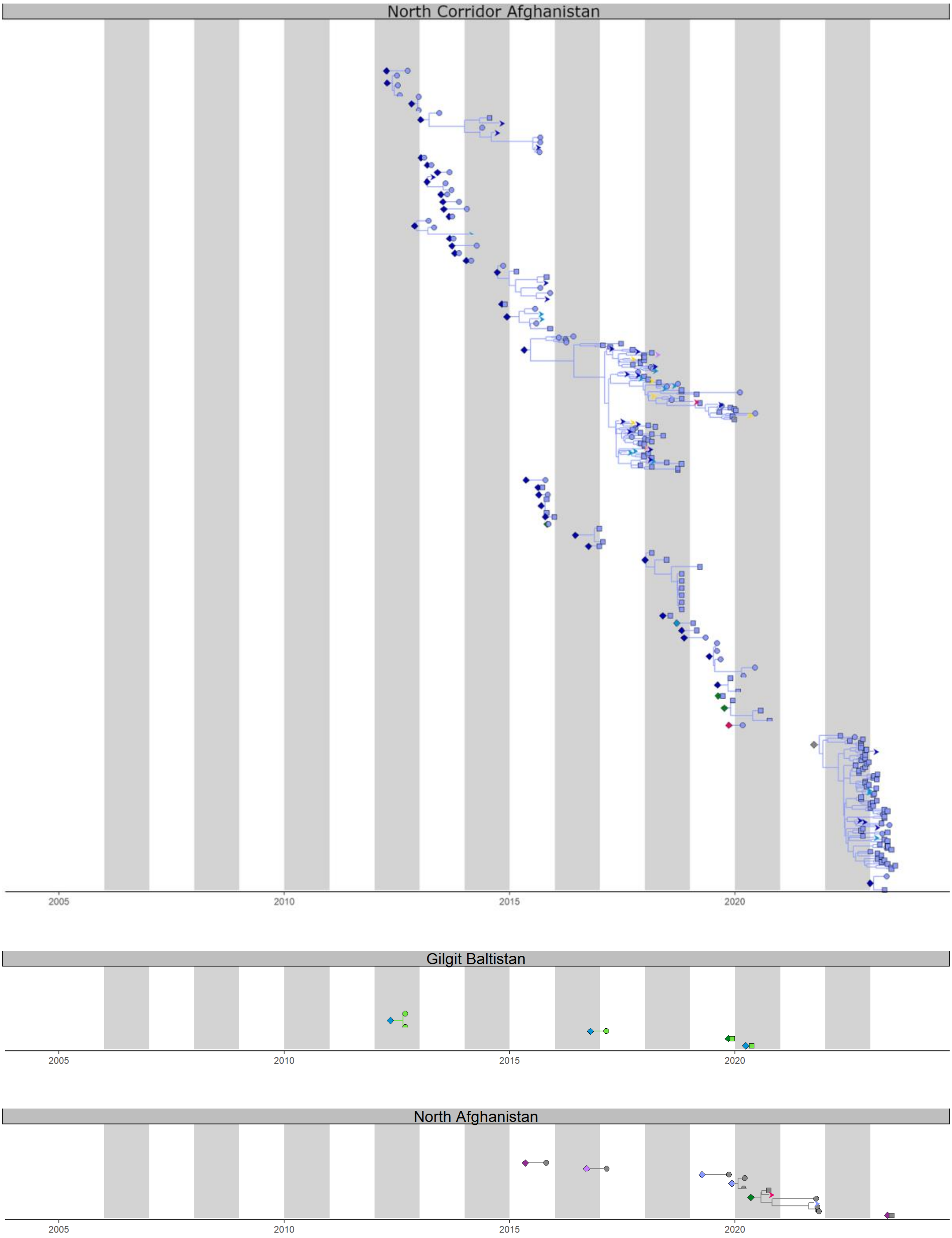

Fig. S6.

Example time-resolved phylogenetic tree of the full dataset split by regions. Here we present the inferred movements on the consensus phylogeny from figure 2, producing one set of LTL plots. In the full analysis this is carried out for a set of posterior phylogenies. A new lineage is plotted after each inferred virus movement between regions. The tips represent sequenced viruses, with round tips representing virus isolates from AFP cases and square tips isolations

from ES. The root of each lineage is annotated with a diamond colored according to the inferred ancestral location. As the time of the virus movement from one region to the other cannot be precisely inferred, the ancestral location is plotted at the mid timepoint of the ancestral branch. Where a lineage leads to onwards transmissions in another region, this is shown with a branch leading to an arrow colored by the region exported to.

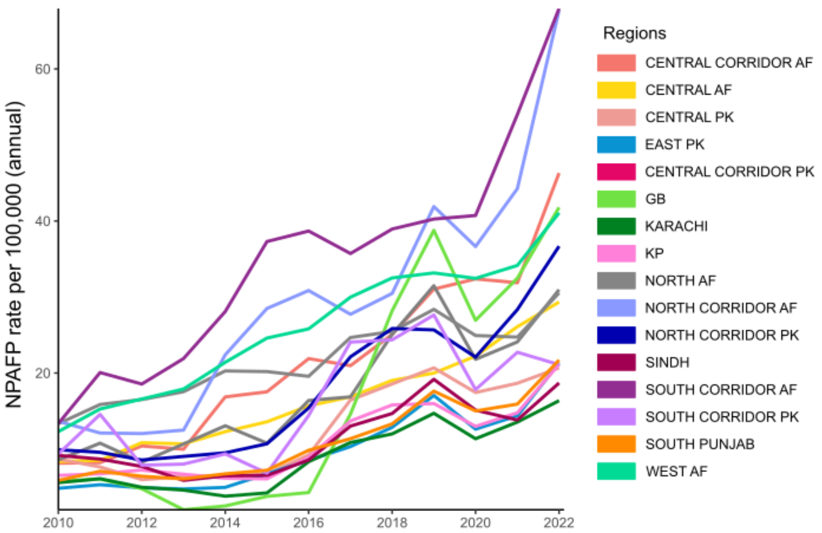

Fig. S7.

Annual non-polio acute flaccid paralysis (NPAFP) rate between 2012 and 2022 across the 16 regions of the polio endemic Pakistan-Afghanistan block. Estimates of population by age group and gender from worldpop.org for 2020 are aggregated to give a denominator of all children under the age of 15 in each region[43]. Annual NPAFP cases in each region from the POLIS database were divided through by the number of children under the age of 15 measured in 100,000s to calculate the NPAFP rate[9].

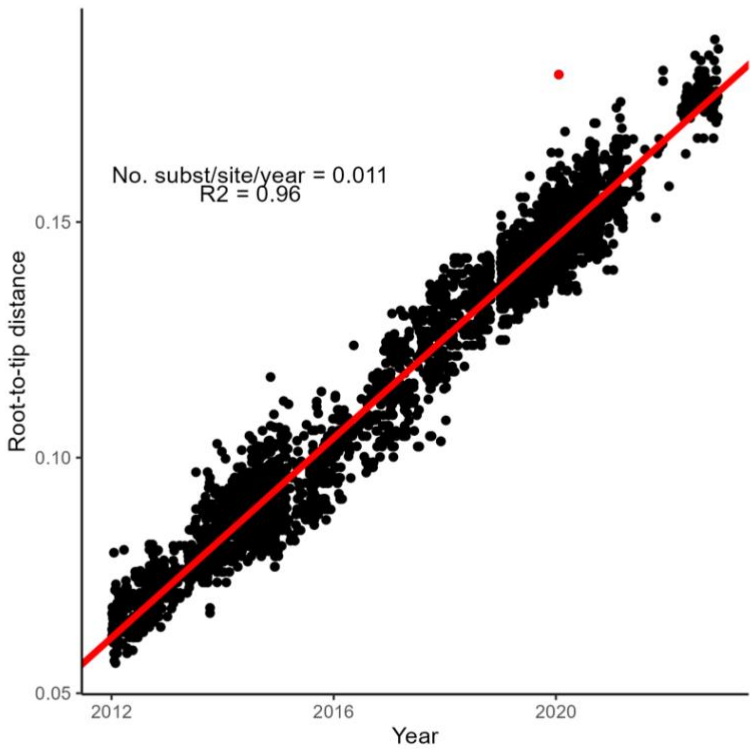

Fig. S8.

Regression of root- to-tip distance on sampling time for the included poliovirus VP1 sequences. The linear trend and high R^2 value suggest that these data are consistent with a strict molecular clock at a substitution rate which aligns with previous reporting. The outlying point highlighted in red was excluded from further analysis.

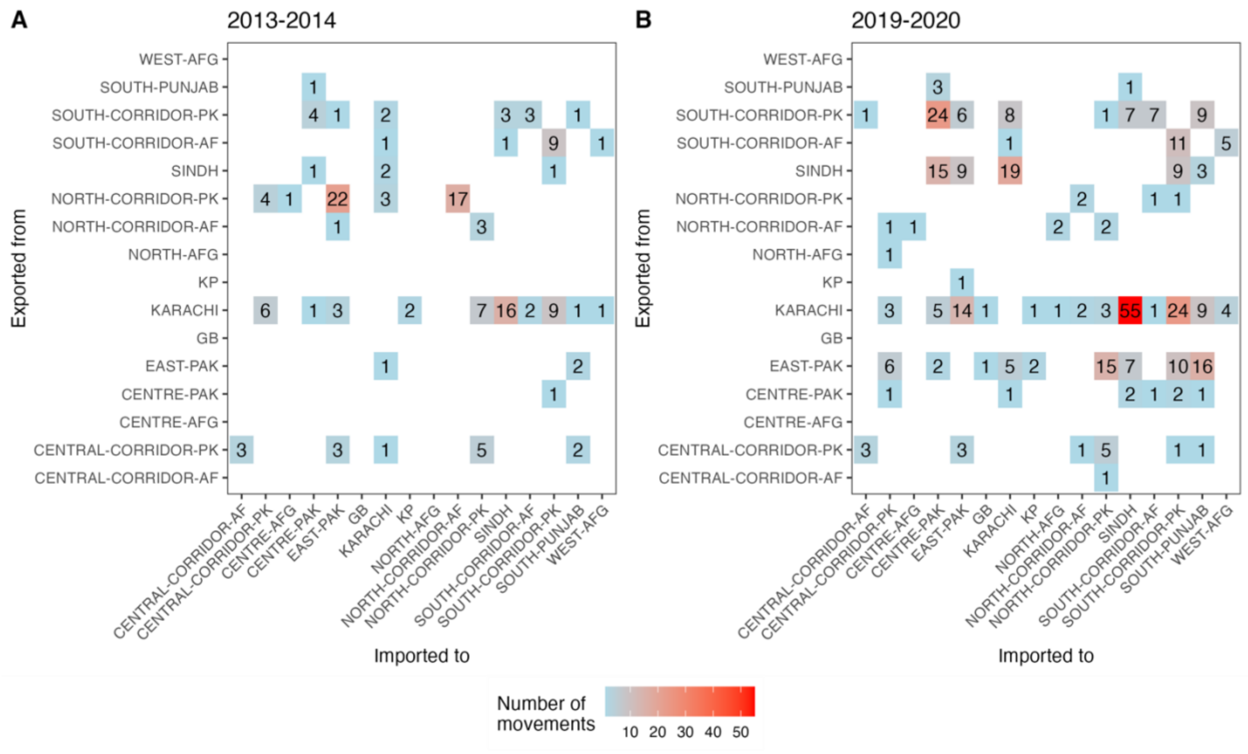

Fig. S9.

Inferred movement events between regions during the A) 2013 to 2014 and B) 2019 to 2020 outbreak periods based on the reconstruction of most likely ancestral regions with sequence data from stool and ES samples using discrete trait analysis. This is an alternative representation of the data plotted in figure 2 of the main text.

Table S1.
Location and sample type metadata for included VP1 sequences. One additional stool sequence was removed from South Punjab as it was an outlier from the molecular clock. Two sequences could not be matched to metadata so were not included in the analysis.

Region	ES	Stool	Total
Central Corridor Af	7	26	33
Central Af	7	3	10
Central Pk	67	32	99
East Pk	512	22	534
Central Corridor Pk	86	326	412
GB	2	3	5
Karachi	592	53	645
KP	1	21	22
North Af	2	8	10
North Corridor Af	143	63	206
North Corridor Pk	357	236	593
Sindh	252	75	327
South Corridor Af	236	145	381
South Corridor Pk	360	58	418
South Punjab	99	22	121
West Af	4	23	27
Total	2727	1116	3843

Table S2.

More detailed correlation between reported cases and reported sequences. Kendall's rank correlation coefficient scores computed for each year and region.

Year	Correlation	Region	Correlation
2008	*No sequence data*	Central Corridor AF	0.92
2009	*No sequence data*	Central AF	*No reported cases*
2010	*No sequence data*	Central PK	0.68
2011	*No sequence data*	East PK	0.21
2012	0.37	Central Corridor PK	0.91
2013	0.43	GB	*No reported cases*
2014	0.71	Karachi	0.19
2015	0.43	KP	1
2016	-0.16	North AF	0.72
2017	0.59	North Corridor AF	-0.11
2018	0.55	North Corridor PK	0.82
2019	0.50	Sindh	0.45
2020	0.18	South Corridor AF	0.57
2021	-0.33	South Corridor PK	0.37
2022	0.33	South Punjab	0.78
2023	1	West AF	1

Table S3.

Numbers of movements into and out of each location inferred under the null (tip-state-swap) and discrete trait models. The bounds represent 95% credible intervals from the Bayesian analysis. The value is the median number of inferred transitions.

	Null model						Discrete trait model					
	Importations			Exportations			Importations			Exportations		
Location	value	lower bound	upper bound	value	lower bound	upper bound	value	lower bound	upper bound	value	lower bound	upper bound
SOUTH-CORRIDOR-AF	425	391	460	261	148	383	36	30	41	60	47	75
WEST-AFG	27	26	27	0	0	1	17	16	19	1	0	3
NORTH-CORRIDOR-AF	208	199	219	22	0	48	46	39	53	56	47	66
CENTRAL-CORRIDOR-AF	33	32	34	0	0	2	13	12	17	2	1	5
CENTRE-AFG	10	10	10	0	0	1	9	9	9	0	0	0
NORTH-AFG	10	10	10	0	0	1	6	6	8	2	0	3
SOUTH-CORRIDOR-PK	474	434	516	368	243	512	125	113	135	123	105	141
SINDH	354	328	382	144	68	231	138	124	152	79	57	100
NORTH-CORRIDOR-PK	656	579	738	1009	798	1211	94	81	107	132	113	150
ENDEMIC-ZONE	467	427	507	353	216	482	42	36	47	47	40	54
EAST-PAK	606	543	672	794	603	1000	137	126	149	127	106	150
SOUTH-PUNJAB	121	117	125	4	0	14	65	61	68	8	4	14
KP	22	22	23	0	0	1	10	9	12	1	0	2
GB	5	5	5	0	0	1	4	4	4	0	0	0
CENTRE-PAK	99	96	102	2	0	9	62	57	67	13	5	21
KARACHI	713	618	808	1264	1032	1479	88	74	102	240	215	268
