# Supplementary material for: Evolution and transmission dynamics of wild poliovirus in Pakistan and Afghanistan (2012-2023)
Source: Nat Commun. 2025 Jun 4;16:5170. doi: 10.1038/s41467-025-60432-x (PMC12137544; doi:10.1038/s41467-025-60432-x)
Supplement: Supplementary file 2 — Description of Additional Supplementary Files [file 41467_2025_60432_MOESM2_ESM.pdf]

**Description of Additional Supplementary Files**

**Supplementary Video 1:** Animated map of viral movements between the included regions through time.
