# Supplementary figures and images for: Evolution and transmission dynamics of wild poliovirus in Pakistan and Afghanistan (2012-2023)
Source: Nat Commun. 2025 Jun 4;16:5170. doi: 10.1038/s41467-025-60432-x (PMC12137544; doi:10.1038/s41467-025-60432-x)

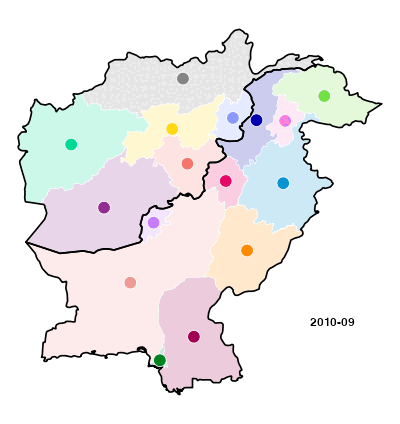

Supplement: Supplementary file 3 — Supplementary Video 1 [file 41467_2025_60432_MOESM3_ESM.gif]
