# Supplementary material for: Evolution and transmission dynamics of wild poliovirus in Pakistan and Afghanistan (2012-2023)
Source: Nat Commun. 2025 Jun 4;16:5170. doi: 10.1038/s41467-025-60432-x (PMC12137544; doi:10.1038/s41467-025-60432-x)
Supplement: Supplementary file 4 — Reporting Summary [file 41467_2025_60432_MOESM4_ESM.pdf]

nature portfolio

Corresponding author(s): David Jorgensen

Last updated by author(s): May 16, 2025

Reporting Summary

Nature Portfolio wishes to improve the reproducibility of the work that we publish. This form provides structure for consistency and transparency in reporting. For further information on Nature Portfolio policies, see our Editorial Policies and the Editorial Policy Checklist.

Statistics

For all statistical analyses, confirm that the following items are present in the figure legend, table legend, main text, or Methods section.

n/a	Confirmed	
☐	☒	The exact sample size (*n*) for each experimental group/condition, given as a discrete number and unit of measurement
☐	☒	A statement on whether measurements were taken from distinct samples or whether the same sample was measured repeatedly
☐	☒	The statistical test(s) used AND whether they are one- or two-sided<br>*Only common tests should be described solely by name; describe more complex techniques in the Methods section.*
☐	☒	A description of all covariates tested
☐	☒	A description of any assumptions or corrections, such as tests of normality and adjustment for multiple comparisons
☐	☒	A full description of the statistical parameters including central tendency (e.g. means) or other basic estimates (e.g. regression coefficient) AND variation (e.g. standard deviation) or associated estimates of uncertainty (e.g. confidence intervals)
☐	☒	For null hypothesis testing, the test statistic (e.g. *F*, *t*, *r*) with confidence intervals, effect sizes, degrees of freedom and *P* value noted<br>*Give P values as exact values whenever suitable.*
☐	☒	For Bayesian analysis, information on the choice of priors and Markov chain Monte Carlo settings
☒	☐	For hierarchical and complex designs, identification of the appropriate level for tests and full reporting of outcomes
☒	☐	Estimates of effect sizes (e.g. Cohen's *d*, Pearson's *r*), indicating how they were calculated

Our web collection on statistics for biologists contains articles on many of the points above.

Software and code

Policy information about availability of computer code

Data collection	This is a secondary analysis of previously collected surveillance data and as such no data collection was carried out.
Data analysis	Analysis was carried out with BEAST v1.10.5, SpreaD3 v0.9.7, MAFFT v7.490, IQ-TREE v1.6.12, and R v4.4.1 code and packages used are available on github and archived with zenodo

For manuscripts utilizing custom algorithms or software that are central to the research but not yet described in published literature, software must be made available to editors and reviewers. We strongly encourage code deposition in a community repository (e.g. GitHub). See the Nature Portfolio guidelines for submitting code & software for further information.

Data

Policy information about availability of data

All manuscripts must include a data availability statement. This statement should provide the following information, where applicable:

- Accession codes, unique identifiers, or web links for publicly available datasets
- A description of any restrictions on data availability
- For clinical datasets or third party data, please ensure that the statement adheres to our policy

Data availability
Epidemiological data analysed in this study were obtained from the World Health Organization (WHO) Polio Information System on 11th April 2024. These data are the property of the individual countries, and data access was provided through the Global Polio Eradication Initiative (GPEI) data sharing agreement. Data are

available from the WHO Institutional Data Access/Ethics Committee for GPEI research partners who meet the criteria for access to confidential data. (https://extranet.who.int/polis/)

The genetic sequence data, accessed on 10th September 2023, are provided by the National Institute of Health, Pakistan (NIH) for this collaborative study. These data are provided under a direct data sharing agreement between NIH Pakistan and Imperial College London. Requests for access to these data can be made directly to NIH at salman14m@gmail.com according to the NIH Pakistan's policy on data sharing using the reference number 1WPV12012308PKAF.

Minimal working example data are provided to reproduce the figures included in the manuscript on GitHub https://github.com/JorgensenD/WPV1_genetics_2023). This does not include any identifiable information or genetic sequence data. This repository is archived with Zenodo63.

Research involving human participants, their data, or biological material

Policy information about studies with human participants or human data. See also policy information about sex, gender (identity/presentation), and sexual orientation and race, ethnicity and racism.

Reporting on sex and gender	n/a
Reporting on race, ethnicity, or other socially relevant groupings	n/a
Population characteristics	n/a
Recruitment	n/a
Ethics oversight	Imperial College Research Ethics Committee

Note that full information on the approval of the study protocol must also be provided in the manuscript.

Field-specific reporting

Please select the one below that is the best fit for your research. If you are not sure, read the appropriate sections before making your selection.

☒ Life sciences ☐ Behavioural & social sciences ☐ Ecological, evolutionary & environmental sciences

For a reference copy of the document with all sections, see nature.com/documents/nr-reporting-summary-flat.pdf

Life sciences study design

All studies must disclose on these points even when the disclosure is negative.

Sample size	No sample size calculation or down-sampling was performed. The analysis is carried out on all genetic sequence data from the region over the time period covered
Data exclusions	Two sequences were excluded as they lacked sufficient metadata to place them in space and time. An additional sequence was found to be an outlier to the well supported molecular clock for poliovirus and so was removed from the analysis. These are outlined in the methods and supplement.
Replication	Bayesian analyses were repeated multiple times to assess convergence Sensitivity analyses were performed to assess the impact fo samplign biases on the inferred outcome variables.
Randomization	Study groupings were based on geographic location. We performed a state-swapping correction to account for differences in sampling intensity by region.
Blinding	Blinding is not relevant to this study. It is a secondary analysis of surveilance data.

Reporting for specific materials, systems and methods

We require information from authors about some types of materials, experimental systems and methods used in many studies. Here, indicate whether each material, system or method listed is relevant to your study. If you are not sure if a list item applies to your research, read the appropriate section before selecting a response.

Materials & experimental systems

n/a	Involved in the study
☒	☐ Antibodies
☒	☐ Eukaryotic cell lines
☒	☐ Palaeontology and archaeology
☒	☐ Animals and other organisms
☒	☐ Clinical data
☒	☐ Dual use research of concern
☒	☐ Plants

Methods

n/a	Involved in the study
☒	☐ ChIP-seq
☒	☐ Flow cytometry
☒	☐ MRI-based neuroimaging

Plants

Seed stocks	n/a
Novel plant genotypes	n/a
Authentication	n/a
